# Development of a core outcome set for physiotherapy trials in adults with bronchiectasis (the COS-PHyBE study): A Delphi study and consensus meeting

**DOI:** 10.1016/j.heliyon.2024.e34101

**Published:** 2024-07-05

**Authors:** Hayat Hamzeh, Carol Kelly, Annemarie L. Lee, Arietta Spinou, Alda Marques, Beatriz Herrero-Cortina, Chris Burtin, Kathleen Hall, Sally Spencer

**Affiliations:** a Faculty of Health, Social Care & Medicine, Edge Hill University, Ormskirk, UK; b Department of Physiotherapy, Monash University, Frankston, 3199, Victoria, Australia; c School of Life Course & Population Sciences, Faculty of Life Sciences & Medicine, King's College London, London, UK; d Respiratory Research and Rehabilitation Laboratory (Lab3R), School of Health Sciences (ESSUA) and Institute of Biomedicine (iBiMED), University of Aveiro, Aveiro, Portugal; e Hospital Clínico Universitario Lozano Blesa, Zaragoza, Spain; f Instituto de Investigación Sanitaria (IIS) de Aragón, Zaragoza, Spain; g Universidad San Jorge, Zaragoza, Spain; h REVAL Rehabilitation Research Center, BIOMED Biomedical Research Institute, Faculty of Rehabilitation Sciences, Hasselt University, Belgium; i Physiotherapy Department, The Prince Charles Hospital, Chermside, Queensland, Australia; j School of Allied Health, Australian Catholic University, Banyo, Queensland, Australia; k Cardio-Respiratory Research Centre, Edge Hill University, Ormskirk, Lancashire, UK; l Health Research Institute, Edge Hill University, Ormskirk, Lancashire, UK

**Keywords:** Bronchiectasis, Physiotherapy, Pulmonary rehabilitation, Core outcome set, Outcome assessment, Delphi method

## Abstract

**Background:**

Physiotherapy is recommended for bronchiectasis management, but there is disparity in evidence supporting its use. This is partly because of inconsistency and poor reporting of outcomes in available studies. A Core Outcome Set (COS) may improve trial consistency and decrease reporting bias. This study aimed to develop a COS for physiotherapy clinical trials in adults with bronchiectasis.

**Methods:**

A comprehensive list of outcomes was developed using a systematic review and qualitative semi-structured interviews with patients with bronchiectasis and physiotherapists.

An international two-round online Delphi survey was conducted. Outcomes scored 7–9 (crucial) by ≥ 70 % of participants and 1–3 (not that important) by ≤ 15 % of participants from each stakeholder in the Likert scale were nominated for inclusion in the COS. Nominated outcomes and those considered crucial by only one of the stakeholders’ groups were further discussed and voted in an international consensus meeting.

**Results:**

A list of 137 outcomes was generated; 104 from literature and 33 from interviews. A Delphi survey containing 59 outcomes was completed by 171 participants from 20 countries. After the consensus meeting, representatives agreed on seven outcomes: health-related quality of life, respiratory symptoms, physical functioning, emotional and psychological functioning, fatigue, adherence to treatment, and functional exercise capacity.

**Conclusion:**

A minimum set of seven outcomes are recommended to be included as measurements in future trials evaluating physiotherapy interventions for bronchiectasis.

## Highlights

• Heterogeneity exists in the outcomes reported in physiotherapy trials for bronchiectasis.
• Core outcome set (COS) is the minimum outcomes agreed to be measured and reported in all trials.
• A complex research design of Delphi study and consensus meeting was used to produce the final COS.
• Physiotherapists, researchers, and patients reached consensus on seven core outcomes.
• Researchers are encouraged to use this COS to improve consistency and optimise synthesis of findings.

## 1 Ethics and consent

The study received ethical approval from the health-related Research Ethics Committee - Edge Hill University (ETH2021-0217). Participant information sheet and consent form was presented before the online survey and a tick box was added to confirm consent.

## 2 Introduction

Bronchiectasis is a chronic respiratory disease characterised by damaged and irreversibly dilated bronchi, causing recurrent infections and hospitalisations [1]. Respiratory physiotherapy is recommended for bronchiectasis management [2] and includes airway clearance therapy (through breathing techniques, positioning options, and clearance devices), endurance and resistance training, physical activity, education, and self-management advice [3,4].Such techniques or strategies may be applied separately or combined in a pulmonary rehabilitation program [5]. There is disparity in recommendations among aspects of physiotherapy for bronchiectasis. While weak recommendations based on low level of evidence were given to airway clearance therapy, strong recommendations based on moderate to high evidence supported exercise [6,7]. This inconsistency is partially due to variability in outcome selection and reporting, making comparison among studies challenging [8]. Core Outcome Set (COS), which defines the minimum set of outcomes that should be measured in all clinical trials for a given condition [9], may be applicable to address this variability. This enables effective evidence synthesis through research consistency and contributes to guideline development, benchmarking physiotherapy, and clinical practice improvement [10]. The need for a COS for physiotherapy research in bronchiectasis has been established [[11], [12], [13]]. A literature review of outcome reporting found high heterogeneity in the use and reporting of outcomes [12], with no single outcome used across all trials and 15.4 % of included outcomes reported only in one trial. Qualitative interviews identified many outcomes not reported in previous trials [13]; such outcomes which are relevant to patients and clinicians may be at risk of being omitted in clinical research.

A COS was previously developed for long-term management of bronchiectasis, which included pharmacological and non-pharmacological interventions [14]. Measurement of 18 outcomes were suggested, nevertheless such a large number of outcomes affects the feasibility of the COS and may hinder uptake in future trials [15]. This COS was developed for general bronchiectasis treatment with limited input from physiotherapists and physiotherapy literature, potentially reducing its relevance in physiotherapy trials. No COS has been developed for physiotherapy interventions in bronchiectasis. As the breadth of research in physiotherapy for bronchiectasis continues to expand, a sound COS is warranted to guide current research. The main aim of this study was to develop a COS specific for use in physiotherapy trials of adults with bronchiectasis.

## 3 Methods

### 3.1 Registration and protocol

The study protocol was prospectively published [11] and was registered in the Core Outcome Measures in Effectiveness Trials (COMET) initiative, available at www.comet-initiative.org (ID: 1931). The study followed the Core Outcome Set-Standards for Reporting (COS-STAR) Statement [16]. The study received ethical approval from the health-related Research Ethics Committee - Edge Hill University (ETH2021-0217).

### 3.2 Research question

What are the minimum set of outcomes that must be included in a physiotherapy core outcome set for adults with bronchiectasis?

### 3.3 Scope

This COS is developed for physiotherapy effectiveness trials involving adults with bronchiectasis, both stable and during exacerbation. It is applicable to trials investigating the following interventions: airway clearance therapy (using expiratory breathing techniques, positioning, or positive expiratory pressure (PEP) devices), endurance and resistance training, physical activity, physiotherapy education and self-management advice. It is also applicable to pulmonary rehabilitation programs which involves combination of physiotherapy treatments but may not be applicable for multidisciplinary pulmonary rehabilitation programs.

### 3.4 Phase one: generating long-list of potential outcomes

Potential outcomes were identified from a systematic search and semi-structured interviews. A systematic review of randomised controlled trials (RCTs) and protocols for RCTs of physiotherapy interventions for adults with bronchiectasis was performed to collect reported outcomes [12]. Outcomes were listed verbatim, then grouped by two reviewers and categorised into domains using the COMET classification [17]. Semi-structured interviews were conducted to identify additional outcomes important to patients who receive any of the treatments described and physiotherapists who assess these outcomes in daily practice [13]. Participants were from four different countries and included experienced physiotherapists working across multiple settings and treating patients with various presentations and needs. A thematic analysis generated a list of outcomes important to patients and clinicians. The two lists of outcomes produced from the systematic review and the interviews were merged and grouped into outcome domains using the COMET taxonomy [10] (Appendix 2).

### 3.5 Phase two: consensus process to prioritise outcomes and finalise COS

Consensus on core outcomes was achieved using a modified Delphi survey followed by a consensus meeting. The survey involved two rounds, allowing participants to rate each item twice, which aided agreement on the most important items [18]. Surveys were conducted using the onlinesurveys.ac.uk interface (JISC, Bristol, UK) to maximise participation and enhance credibility [19].

### 3.6 Sample and recruitment

Three stakeholders' groups were invited to participate [1]: patients: adults >18 years old with bronchiectasis [2], clinicians: physiotherapists involved in bronchiectasis care, and [3] researchers: researchers involved in clinical trials of physiotherapy for bronchiectasis. Participants able to complete a survey in the English language were included. There is no agreement on adequate sample size for Delphi studies, but good representation of each group is essential [10,19], A sample of 12 participants is suggested for consensus meetings as it allows for comprehensive discussion to take part [20,21].

Recruitment was through multiple channels to maximise international participation and representation of various demographic populations. Recruitment adverts were sent to patient networks (such as the European Lung Foundation), professional organisations (such as the International Confederation of Cardiorespiratory Physical Therapists (ICCrPT)), research networks (such as the European Multicentre Bronchiectasis Audit and Research Collaboration (EMBARC)), and other professional organisations and societies (Appendix 1). Adverts were also posted on patient groups and forums on social media, suggested by patient representatives. Researchers, identified during the literature review, were invited via email. Twitter account of the study, @PhyBEStudy, was also used to recruit participants.

### 3.7 Formulation of the survey items

An international steering committee of seven expert physiotherapists and three patients was formed to oversee the Delphi study process. The list of outcomes produced in phase one was sent to the committee for discussion and feedback. Similar outcomes were combined and the list of outcomes for the Delphi survey was decided. Outcomes from each domain were presented separately, and a definition for each outcome in lay language was prepared then verified by two patient representatives. An online survey was created, and pilot tested to ensure clarity, feasibility, and technical performance before data collection (Appendix 3). Participant information sheet and consent form was presented before the online survey and a tick box was added to confirm consent.

### 3.8 Delphi survey: round one (R1)

Participants were asked to rate each outcome's importance according to their own experiences and opinions. A nine-point Likert scale was used to score items according to their importance, and an option of 'unable to score' was added for each item. A score of 1–3 signified an outcome of limited importance, 4 to 6 important but not critical, and 7 to 9 critical. This scoring system is widely adopted for agreement studies [20,22] and is recommended by the COMET [9]. An open-ended question was added for the participant to suggest any other outcomes, and those were added to the second round if they were not already included in the survey. The survey was open from June 1, 2022 to July 2, 2022. Weekly email reminders were sent to encourage participation.

### 3.9 Delphi survey: round two (R2)

Participants who completed R1 were sent invitations to complete R2, which was open from July 15, 2022 to August 31, 2022. All outcomes were carried through to R2, in addition to newly suggested outcomes from the open-ended question in R1. Percentages of scores obtained from each stakeholder group in R1 were displayed with corresponding outcomes (Appendix 4). Participants were asked to consider previous group scores and rate each item using the same Likert scale. Weekly email reminders were sent to encourage participation.

Data analyses were performed and presented separately for each stakeholder group to allow comparisons. Response rate was calculated by comparing number of participants between both rounds. If a participant did not complete all items, available responses were included in analysis.

Percentages of participants’ scores for each item in R2 were used to determine consensus, based on the pre-specified criteria published in the protocol [11] and based on COMET suggestions [10]. The following consensus criteria were applied: Outcomes with a score of 7–9 from more than 70 % of participants and a score of 1–3 from less than 15 % of participants in all stakeholder groups were nominated for inclusion. Outcomes with a score of 7–9 from less than 50 % of participants in all groups were excluded from the final COS.

### 3.10 Consensus meeting

Following the Delphi survey, a consensus meeting was held to discuss results and vote on the final COS outcomes. The meeting was attended by a group of expert physiotherapists (both clinically and in research) and a group of patients. Participants were purposefully sampled from R2 to ensure balanced numbers of patients and physiotherapists attending the meeting [23].

Before the meeting, people who agreed to attend received a list of outcomes to be discussed with their definitions, their own R2 scoring, and an electronic informed consent form. They also received a video explaining the study, core outcome sets, and the outcome selection process. A detailed explanation on meeting expectations, the voting process, and technical information about the software was provided.

The meeting was held online using Microsoft Teams. Participants could use the chat function to express their opinions. The facilitator (HH) used a pre-defined meeting agenda, which was agreed by the Delphi study committee. Participants were offered a pre-meeting to test the technology and ask any questions if needed.

The main aim of the meeting was to reduce the number of outcomes that met the inclusion criteria after R2 (n = 36). During discussion, participants were encouraged to distinguish between outcomes applicable to all physiotherapy trials and outcomes that are important but should not always be measured. It was emphasised that the COS should contain outcomes to be measured in all future research, and that outcomes not included are still important and can be measured as additional outcomes. All participants were encouraged to actively engage in the discussion, and contrasting views were sought.

Following discussion, participants voted on items to be included in the final COS. The Microsoft Teams polling function was used for voting, and voting results were displayed live. When participants faced technical difficulties with the voting process, they were asked to submit their vote via the chat function or verbally during the voting process.

Stricter criteria were needed to reduce the number of outcomes during the consensus meeting. Therefore, a three-point scale was used instead of the nine-point scale, as this has been shown to help participants discriminate further and results in a lower number of outcomes while not missing core measures [24]. Participants were asked to score each outcome as [1]: 'not important enough and should be excluded from the COS' [2], 'important but not critical to be included in the COS', and [3] 'critical and should be included in the COS'. Voting results were calculated for all participants and separately for the patients' and physiotherapists' groups. Items scored as critical by more than 70 % of all participants were included in the final COS. As some voting was not conducted using the voting function due to technical issues, the final COS could not be ratified during the meeting. Final voting results were calculated and revised, then emailed to all participants to review. All participants agreed on the final COS. After the meeting, all participants were asked to fill out an online form – provided by COMET - to provide their feedback on the consensus meeting [25].

### 3.11 Deviations from protocol

It was decided in the protocol that outcomes achieving criteria for inclusion will be included in the final COS, while outcomes achieving no consensus will be taken to the consensus meeting for discussion and voting. As many outcomes were voted for inclusion during R2 (n = 36), those outcomes were taken to the consensus meeting with the aim of item reduction. A decision was made to exclude items which achieved no consensus (n = 23) during R2 without further voting. It was not possible to provide participants with their own individual scores during R2, because this feature was not available in the software used. This was replaced with group scores for each of the three stakeholder groups (patients, clinicians, and researchers) alongside charts explaining scores. During the consensus meeting, participants were grouped into two groups instead of three, patients and physiotherapists. This is unlikely to have affected consensus as most physiotherapists who identified as either a clinician or researcher were involved in both activities.

## 4 Results

### 4.1 Initial list of outcomes

**Fig. 1:**
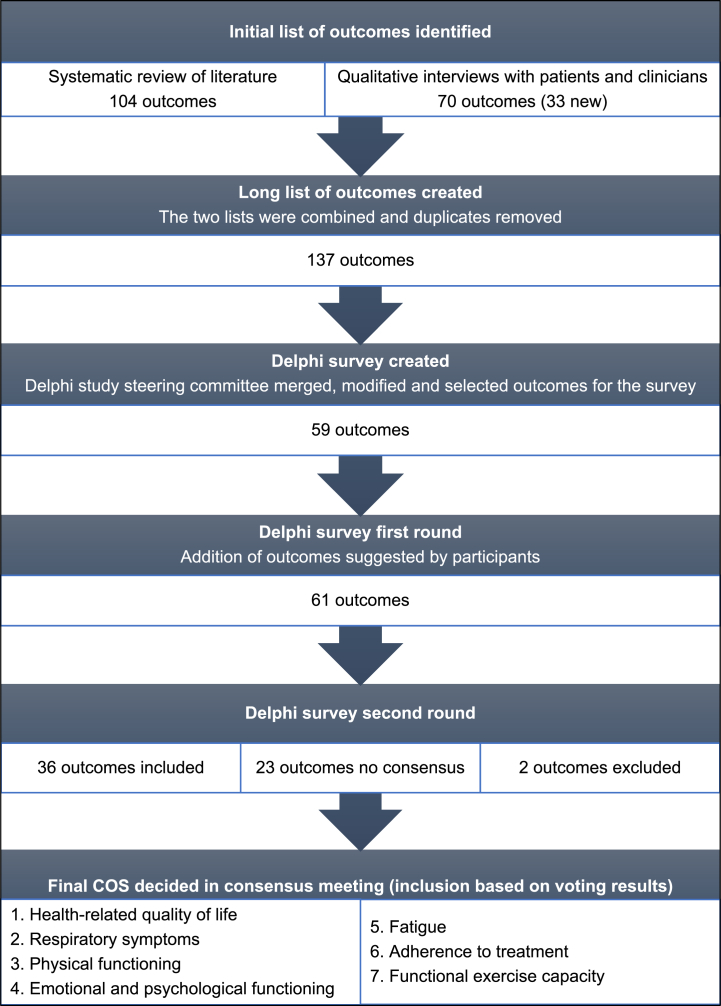
Overview of the COS development process and main results.

An overview of outcomes identified in all stages of the COS-PHyBE study is explained in Fig. 1. Three-hundred and thirty-one verbatim outcome terms were identified from the systematic review, which included 37 trials (1202 participants) and 17 trial protocols [12]. A list of 104 unique outcomes classified into 23 domains was created. An additional 33 outcomes of importance to patients and physiotherapists were elicited through the 18 semi-structured qualitative interviews and not identified in the systematic review [12], Collectively, a list of 137 outcomes was generated and taken into the next phase to establish consensus on the core outcomes (Appendix 2). After several rounds of email communication, the COS-PHyBE study committee agreed to include 59 outcomes in the Delphi study (Appendix 3).

### 4.2 Delphi survey

Round one was completed by 171 participants: 86 (50.3 %) patients, 39 (22.8 %) physiotherapists, and 49 (28.7 %) researchers. Participants were from 20 countries; the highest recruitment location for all participants was the UK (38.0 %), followed by Europe (29.8 %), and Oceania (17.5 %), with the majority of respondents from high income countries (94.2 %). In total, 137 (80.1 %) respondents were females, while 34 (19.9 %) were males. Detailed demographics of participant groups are displayed in Table 1, Table 2.

**Table 1:** Characteristics of patient participants.

Patients		Round 1	Round 2
**Number (% of total participants)**		86 (50.3 %)	68 (79.1)
**Female (%)**		73 (84.9 %)	59 (86.8 %)
**Age mean (SD)**		63.2 (13.9)	64.3 (12.6)
**Region**	UK	48 (55.8 %)	36 (52.9 %)
	Europe	7 (8.1 %)	7 (10.3 %)
	Oceania	9 (10.5 %)	8 (11.8 %)
	Americas	20 (23.3 %)	16 (23.5 %)
	Middle East and Africa	2 (2.3 %)	1 (1.5 %)
Economy *	High-Income	85 (98.8 %)	68 (100 %)
	Upper-Middle-Income	0 (0.0 %)	0 (0.0 %)
	Lower-Middle Income	1 (1.2 %)	0 (0.0 %)
**Highest level of Education**	Primary education	1 (1.2 %)	1 (1.5 %)
	Secondary education	20 (23.3 %)	17 (25.0 %)
	Undergraduate education	55 (64.0 %)	44 (64.7 %)
	Other	10 (11.6 %)	6 (8.8 %)
**Years since diagnosis**	<5	24 (27.9 %)	15 (22.1 %)
	5–10	21 (24.4 %)	18 (26.5 %)
	11–20	16 (18.6 %)	14 (20.6 %)
	21–30	7 (8.1 %)	6 (8.8 %)
	>30	18 (20.9 %)	15 (22.1 %)
**Exacerbation frequency (previous 12 months)**	None	30 (34.9 %)	25 (36.8 %)
	1–4	39 (45.3 %)	30 (44.2 %)
	≥5	19 (22.1 %)	13 (19.1 %)
**Hospitalisations (previous 12 months)**	None	69 (80.2 %)	56 (82.4 %)
	1–4	17 (19.8 %)	12 (17.7 %)
	≥5	3 (3.5 %)	0 (0.0 %)
**Antibiotic courses (previous 12 months)**	None	23 (26.7 %)	20 (29.4 %)
	1–4	45 (52.3 %)	35 (51.4 %)
	≥5	19 (22.1 %)	13 (19.1 %)
**ICU admissions (previous 12 months)**	No	84 (97.7 %)	67 (98.5 %)
	Yes	2 (2.3 %)	1 (1.5 %)
**Urgent healthcare visits (previous 12 months)**	None	35 (40.7 %)	30 (44.1 %)
	1–4	37 (43.1 %)	29 (42.6 %)
	≥5	16 (18.6 %)	9 (13.2 %)
Type of physiotherapy (% using) *	ACT breathing techniques	71 (82.6 %)	55 (80.9 %)
	ACT devices	68 (79.1 %)	52 (76.5 %)
	ACT body positions	43 (50.0 %)	37 (54.4 %)
	ACT manual techniques	33 (38.4 %)	27 (39.7 %)
	Self-management advice	36 (41.9 %)	30 (44.1 %)
	Pulmonary rehabilitation	18 (20.9 %)	16 (23.5 %)
	Exercise and physical activity	70 (81.4 %)	57 (83.8 %)
	Physiotherapy adjunct treatment	40 (46.5 %)	42 (61.8 %)
	Other	7 (8.1 %)	2 (2.9 %)
**Received physiotherapy while hospitalised**		39 (45.3 %)	32 (47.1 %)
**Participated in research**		6 (7.0 %)	5 (7.4 %)

*Economy of participants' country, according to the World Bank Classification [26].

ICU: Intensive Care Unit.

* ACT = Airway clearance techniques.

•Airway clearance breathing techniques (e.g., active cycle of breathing techniques, autogenic drainage, huffing).

•Airway clearance devices (e.g., Positive expiratory pressure (PEP), Oscillating positive expiratory pressure (O-PEP), High-Frequency Chest Wall Oscillation Vest, Intrapulmonary percussive ventilation (IPV)).

•Airway clearance using body positions (e.g., postural drainage, ELTGOL).

•Airway clearance using manual techniques (e.g., manual percussion, shaking, vibration).

•Self-management advice, and education provided by a physiotherapy specialist.

•Pulmonary rehabilitation.

•Exercise and physical activity.

Physiotherapy adjunct treatment (Mucoactive drugs (e.g., isotonic saline, hypertonic saline, mannitol).

**Table 2:** Characteristics of physiotherapists clinicians and researchers' participants.

	Physiotherapists		Researchers	
	Round 1	Round 2	Round 1	Round 2
**Number (% of total participants)**	39 (22.8 %)	21 (58.3 %)	46 (26.9 %)	30 (61.2 %)
**Female (%)**	34 (87.2 %)	21 (100 %)	30 (65.2 %)	18 (60 %)
**Country**				
**UK**	7 (17.9 %)	7 (33.3 %)	10 (21.7 %)	9 (30.0 %)
**Europe**	15 (38.5 %)	4 (19.0 %)	29 (63.0 %)	15 (50.0 %)
**Oceania**	16 (41.0 %)	9 (42.9 %)	5 (10.9 %)	4 (13.3 %)
**Americas**	0 (0.0 %)	0 (0.0 %)	1 (2.2 %)	1 (3.3 %)
**Middle East and Africa**	1 (2.6 %)	1 (4.8 %)	1 (2.2 %)	1 (3.3 %)
Economy *				
**High-Income**	38 (97.4 %)	20 (95.2 %)	38 (82.6 %)	24 (80.0 %)
**Upper-Middle-Income**	0 (0.0 %)	0 (0.0 %)	7 (15.2 %)	5 (16.7 %)
**Lower-Middle Income**	1 (2.6 %)	1 (4.8 %)	1 (2.2 %)	1 (3.3 %)
**Highest level of Education**				
**Undergraduate university**	13 (33.3 %)	8 (38.1 %)	0 (0.0 %)	0 (0.0 %)
**Cardiorespiratory PT postgraduate**	11 (28.2 %)	6 (28.6 %)	17 (37.0 %)	10 (33.3 %)
**Postgraduate MSc level**	14 (35.9 %)	7 (33.3 %)	10 (21.7 %)	7 (23.3 %)
**PhD and doctoral level**	1 (2.6 %)	0 (0.0 %)	19 (41.3 %)	13 (43.3 %)
**Years of experience Mean (SD)**	17.8 (8.8)	19.6 (8.3)	17.7 (8.0)	18.7 (8.5)
**Years of bronchiectasis experience Mean (SD)**	11.4 (7.5)	14.6 (7.3)	11.8 (7.0)	12.0 (7.3)
**Primary employment setting (% using)**				
**Hospital/inpatient**	21 (53.8 %)	9 (42.9 %)	23 (50.0 %)	14 (46.7 %)
**Hospital/outpatient**	18 (46.2 %)	11 (52.4 %)	17 (37.0 %)	11 (36.7 %)
**Private physiotherapy clinic**	9 (23.1 %)	4 (19.0 %)	3 (6.5 %)	2 (6.7 %)
**Community physiotherapy service**	6 (15.4 %)	5 (23.8 %)	2 (4.3 %)	1 (3.3 %)
**Research/academic institution**	0 (0.0 %)	0 (0.0 %)	29 (63.0 %)	20 (66.7 %)
**Patient organisation**	0 (0.0 %)	0 (0.0 %)	1 (2.2 %)	1 (3.3 %)
**Bronchiectasis patients assessed weekly**				
**None**	0 (0.0 %)	0 (0.0 %)	5 (10.9 %)	3 (10.0 %)
**1–10**	36 (92.3 %)	19 (48.7 %)	32 (69.6 %)	21 (70.0 %)
**10**–**20**	1 (2.6 %)	0 (0.0 %)	7 (15.2 %)	5 (16.7 %)
**20**–**30**	1 (2.6 %)	1 (2.6 %)	1 (2.2 %)	0 (0.0 %)
**30**–**40**	1 (2.6 %)	1 (2.6 %)	0 (0.0 %)	0 (0.0 %)
**40**–**50**	0 (0.0 %)	0 (0.0 %)	1 (2.2 %)	1 (3.3 %)
**Research activity (% using)**				
**Designing research**	3 (7.7 %)	1 (4.8 %)	31 (67.4 %)	24 (80.0 %)
**Conducting research**	7 (17.9 %)	6 (28.6 %)	35 (76.1 %)	23 (76.7 %)
**Systematic reviews**	6 (15.4 %)	2 (9.5 %)	18 (39.1 %)	14 (46.7 %)
**Developing guidelines**	3 (7.7 %)	1 (4.8 %)	15 (32.6 %)	14 (46.7 %)
**Clinical audit or quality control and improvement**	16 (41.0 %)	11 (52.4 %)	8 (17.4 %)	8 (26.7 %)
**Not involved in research**	18 (46.2 %)	9 (42.9 %)	0 (0.0 %)	0 (0.0 %)

Participants suggested 42 additional outcomes at R1. Upon reviewing these, two new outcomes: sexual function and general health status were added to R2 items. The remaining suggestions were excluded because they were included in other outcomes (n = 25), were not relevant (n = 12), or were outcome measurement instruments (n = 3).

The second round was completed by 119 participants. Response rate in R2 was 69.6 % for all participants; 79.1 % patients, 61.2 % researchers, and 58.3 % clinicians. Only two outcomes met the exclusion criteria based on R2, while 23 achieved no consensus, and 36 achieved the criteria for inclusion (Appendix 5). Therefore, those which met the inclusion criteria were prioritised for discussion in the consensus meeting, with the aim of COS outcome reduction. Inspection of the distribution of R1 participant average outcome rating did not reveal differences between those who did or did not complete both rounds (Appendix 6).

### 4.3 Consensus meeting results

Twenty-two participants, 12 patients and 10 physiotherapists agreed to attend the meeting, with 15 participants, 7 patients and 8 physiotherapists attending. All professionals who attended were physiotherapists with expertise in both clinical practice and research.

At the end of voting, eight outcomes achieved the criteria to be included in the final COS i.e.: more than 70 % from all meeting participants (Appendix 7). Following discussion, the panel agreed that the outcome' activities of daily living' can be measured within the outcome' physical functioning'. Therefore, it was deleted, and the definition of physical functioning outcome updated to include activities of daily living. Fifteen outcomes were scored >70 % by patients but did not achieve the criteria for inclusion, while only one outcome (cough) was scored >70 % by professionals and did not achieve the criteria for inclusion.

The final COS (Table 3) included seven outcomes to be measured in all respiratory physiotherapy trials: health-related quality of life (HRQoL), respiratory symptoms, physical functioning, emotional and psychological functioning, fatigue, adherence to treatment, and functional exercise capacity (to be measured only if the intervention includes an endurance or resistance exercise component).

**Table 3:** Description of the Core Outcome Set for physiotherapy trials in bronchiectasis.

Outcome included in the final COS	Description	R2 (Patients)	R2 (Clinicians)	R2 (Researchers)	Consensus Meeting
**Health-related quality of life**	An overall evaluation of how a person's health affects their life and general wellbeing, to be reported as a single score.	96 %	100 %	97 %	93 %
**Respiratory symptoms**	Burden of respiratory symptoms, such as cough, sputum, and breathlessness.	99 %	95 %	97 %	80 %
**Physical functioning**	General evaluation of ability to function physically, e.g., mobility, transfers, activities of daily living, sleeping.	96 %	100 %	97 %	93 %
**Emotional and psychological functioning**	General evaluation of emotional and psychological wellbeing, e.g., anxiety and depression.	94 %	90 %	77 %	100 %
**Fatigue**	Intensity, frequency, and duration of fatigues. Defined as feeling tired and having no or low energy.	88 %	95 %	90 %	71 %
**Adherence to treatment**	Measuring whether the patient is applying the physiotherapy program using the required number of applications and correct method.	94 %	100 %	97 %	80 %
**Functional exercise capacity**	Ability to perform aerobic activity under controlled conditions, e.g., field walking capacity.	91 %	100 %	97 %	92 %

R2 = Delphi round two.

The meeting feedback was completed by 80 % of participants (Appendix 8). All participants reported satisfaction with the information provided before meeting and were comfortable communicating their views during the meeting. Four patients were neutral regarding the fairness of results; they explained that they felt more important outcomes to patients should be included, since some achieved 100 % by patient participants but were excluded by professionals' voting.

## 5 Discussion

This international Delphi study developed a COS of seven outcomes to be used in future research for physiotherapy in bronchiectasis. A panel of international representatives agreed that these were the critical outcomes that should be measured in all future trials of respiratory physiotherapy for bronchiectasis. This COS is applicable for trials investigating any respiratory physiotherapy technique in bronchiectasis, including exercise and physical activity whether tested in isolation or as a part of a comprehensive physiotherapy program. However, it may not be applicable for pulmonary rehabilitation programs which involves multidisciplinary management or a sample of multiple respiratory conditions.

Results from this project will inform future research by improving homogeneity of outcomes used, facilitating the combination of trial results in meta-analyses and clinical guidelines. Thus, it is seen as a valuable way of decreasing research waste [27]. Using the outcomes suggested in this COS in trials will also ensure outcomes important to patients and clinicians are consistently used in research. Thus, endorsement of research by patients and public may be improved [8]. Uptake of this COS is not meant to restrict outcome reporting, as trialists may measure additional outcomes to the COS based on their individual trial's aims.

Outcomes included in this COS reflect the nature of respiratory physiotherapy, focusing on the effect of treatment on patient lives rather than clinical and/or physiological outcomes. An overlap is evident in the outcomes of the current COS and the outcomes recommended by two projects involving physiotherapy in COPD [28] and for critical care survivors [29]. Those outcomes are HRQoL, physical functioning, and functional exercise capacity. This is not surprising as physiotherapy practice have similar goals across these conditions. The outcomes of HRQoL, adherence to treatment, and exercise tolerance were previously prioritised in a broader COS for bronchiectasis [14] in addition to their inclusion here. Other outcomes previously recommended [14] were identified as important during this study but were not selected as part of the final set of outcomes. This overlap in outcomes could further promote consistency in outcome measurement. Having multiple COS for the same condition may facilitate outcome selection in trials based on the type of intervention used.

In the consensus meeting, some outcomes did not achieve the criteria for inclusion in the final COS although they scored higher than 80 % level of agreement by patients (Appendix 8). A small number of items were required for the COS to be feasible, while ensuring the outcomes chosen are relevant to both patients and researchers. Therefore, only items which achieved highly by both groups attending the meeting were included. Outcomes of importance to patients which did not reach the threshold for inclusion in the COS could be used as additional or secondary outcomes. They may also be used to guide future research in bronchiectasis into patient-focussed issues. Periodic revisions of the COS will determine whether any change of the outcomes currently included is needed [10].

Lack of validated instruments for assessing some outcomes in the context of a bronchiectasis trial may have discouraged their selection by participant researchers. Multiple tools and instruments are available to measure those outcomes prioritised in this COS, including HRQoL tools developed and/or validated in bronchiectasis population [30]. Some outcomes included in the COS, i.e., respiratory symptoms, physical functioning, emotional and psychological functioning, and fatigue, can be measured individually and/or are included within selected HRQoL tools [[31], [32], [33]]. Their inclusion as separate outcomes in the COS indicate individual reporting of their scores notwithstanding the method of measurement. Future research should assess the quality of specific tools in bronchiectasis and propose those that are most valid and reliable to accompany this COS, alongside any modifications or development of new tools. Development of this core measurement set is the next planned phase of the COS-PHyBE study [11].

## 6 Strengths and Limitations

The COS-PHyBE study presents a focused COS developed for physiotherapy trials including adults with bronchiectasis, and can be easily applied in future trials. The study followed robust methodology based on the COMET guidelines, including a pre-published protocol to ensure validity of the results [11] and quality indicators based on the published COS-STAR standards [16]. This COS is applicable to future trials investigating any type of respiratory physiotherapy in bronchiectasis. This is due to the inclusion of patients receiving all types of respiratory physiotherapy and clinicians working across broad settings of practice (Table 1, Table 2). Additionally, the literature review included outcomes from trials which investigated any type of respiratory physiotherapy for bronchiectasis. The study demonstrated a low attrition rate between the Delphi rounds and positive feedback was received for the consensus meeting. The methods used in this study ensured all opinions were considered, and the final COS was relevant to both patients and professionals.

Participants were from 20 countries, but with limited participation from lower income countries in Asia, Africa, and the Middle East. This disproportionality is a recognised issue in COS research where inclusion of participants from low and middle-income countries (LMICs) is lacking [34], which may affect the international generalisability and uptake of the COS [10]. However, geographical distribution of participants in this study reflects the geographical sources of available research, as evident in the systematic review [12], where majority of trials were conducted in Europe and Australia. Therefore, this COS is considered relevant to areas of the world where further research is expected. Additionally, data on bronchiectasis from low and middle-income countries is scarce according to available reports [35,36]. Inclusion of LMICs will be sought in future updates of the COS when more LMICs bronchiectasis data becomes available. Translating the survey into languages other than English could have increased the response rate from non-English speaking countries.

It was not possible to exclude several items based on the results of R2 Delphi survey. This was notably because the patient group deemed most of the outcomes as extremely important, resulting in large number of items to be discussed and voted on during the consensus meeting. Reducing the number of included outcomes was a priority for the meeting. Therefore, a more stringent voting system of three points was used. The importance of agreeing on a small number of items in the COS was shared with the participants before the meeting and emphasised again during the discussion. In addition, the difference between important outcomes (to be measured in research but not necessarily all studies) and core outcomes (which should be measured in all future studies) was explained.

## 7 Conclusion

The COS-PHyBE study proposed seven outcomes relevant to patients, physiotherapists, and researchers for inclusion in future trials of physiotherapy in bronchiectasis. Uptake of this COS will improve the potential for comparisons to take place across trials.

## CRediT authorship contribution statement

**Hayat Hamzeh:** Writing – review & editing, Writing – original draft, Project administration, Methodology, Funding acquisition, Formal analysis, Data curation, Conceptualization. **Carol Kelly:** Writing – review & editing, Validation, Supervision, Methodology, Conceptualization. **Annemarie L. Lee:** Writing – review & editing, Validation, Resources, Data curation. **Arietta Spinou:** Writing – review & editing, Validation, Data curation. **Alda Marques:** Writing – review & editing, Validation, Data curation. **Beatriz Herrero-Cortina:** Writing – review & editing, Validation, Data curation. **Chris Burtin:** Writing – review & editing, Validation, Data curation. **Kathleen Hall:** Writing – review & editing, Validation, Data curation. **Sally Spencer:** Writing – review & editing, Validation, Supervision, Methodology, Formal analysis, Conceptualization.

## Declaration of competing interest

The authors declare that they have no known competing financial interests or personal relationships that could have appeared to influence the work reported in this paper.

## Footnotes

Appendix A Supplementary data to this article can be found online at https://doi.org/10.1016/j.heliyon.2024.e34101.
