# Supplementary material for: Development of a core outcome set for physiotherapy trials in adults with bronchiectasis (the COS-PHyBE study): A Delphi study and consensus meeting
Source: Heliyon. 2024 Jul 5;10(14):e34101. doi: 10.1016/j.heliyon.2024.e34101 (PMC11292240; doi:10.1016/j.heliyon.2024.e34101)
Supplement: Multimedia component 1 [file mmc1.docx]

## Supplementary file

**Appendix 1: Delphi Study recruitment strategy**

- - - 1. **Patient advocacy groups contacted for Delphi recruitment**

European Lung Foundation (ELF)

Asthma and lung UK (British Lung foundation)

Bronchiectasis NTM 360 (COPD foundation)

Lung foundation Australia (LFA)

Respiratory Voices Network (Asthma and lung UK)

NTM Australian Support Group

Asthma Lung UK (ALUK) patient forum

NTM research and support

PhysioAssist

Canadian bronchiectasis support group

- - - 1. **Professional bodies and interest groups contacted for Delphi recruitment**

The European Multicentre Bronchiectasis Audit and Research Collaboration Network (EMBARC)

The Association of Chartered Physiotherapists in Respiratory Care (ACPRC)

Primary Care Respiratory Society (PCRS)

The International Confederation of Cardiorespiratory Physical Therapists (ICCrPT)

Pulmonary rehabilitation assembly

European respiratory society (ERS)
The Academy of Cardiovascular & Pulmonary Physical Therapy - APTA-CVP

Spanish Society of Pneumology and Thoracic Surgery (SEPAR)

Spanish Association of Physiotherapists (AEF)

- - - 1. **Social media and online platforms used for Delphi recruitment**

Twitter page @phybestudy

Bronchiectasis support group (Facebook)

Lung matters (Facebook group)

Bronchiectasis R us online forum (http://www.bronchiectasis.info/)

- - - 1. **Other**

Personal email invitations to 270 researchers identified through literature search.

Contacts of the research team.

**Appendix 2: The initial list of outcomes (additional outcomes from qualitative study are in bold)**

| Core area COMET | Domain | Outcome |
| --- | --- | --- |
| **Mortality/survival** | **1. Mortality/survival** | 1. **Death from any cause** |
|  |  | 1. **Death from bronchiectasis complications** |
| Clinical/Physiological outcomes | Disease activity | 1. Exacerbation frequency |
|  |  | 1. Time to first exacerbation |
|  |  | 1. **Disease progression** |
|  |  | 1. Disease severity |
|  | 2. Blood and lymphatic system outcomes | 1. Blood cell count |
|  | 3. Cardiac outcomes | 1. Heart rate |
|  |  | 1. **Blood pressure** |
|  | 9. General outcomes | 1. Body weight |
|  |  | 1. Body composition |
|  | 12. Infection and infestation outcomes | 1. Sputum neutrophil elastase level |
|  |  | 1. Nasal lavage fluid |
|  |  | 1. Exhaled breath condensate |
|  |  | 1. Cytokines in Exhaled breath condensate |
|  |  | 1. Ph level in Exhaled breath condensate |
|  |  | 1. Exhaled Nitric Oxide Fraction (FENO) |
|  |  | 1. Cytokine level in nasal lavage fluid |
|  |  | 1. TNF-α in nasal lavage fluid |
|  |  | 1. IL-6 and IL-10 in nasal lavage fluid |
|  |  | 1. Blood inflammation markers |
|  |  | 1. Sputum inflammation markers |
|  | 15. Musculoskeletal and connective tissue outcomes | 1. Muscle strength |
|  | 22. Respiratory, thoracic, and mediastinal outcomes | 1. Oxygen saturation SPO2 |
|  |  | 1. Airway resistance |
|  |  | 1. Respiratory rate |
|  |  | 1. Respiratory resistance |
|  |  | 1. Lung sounds |
|  |  | 1. Non-invasive ventilation resistance rate |
|  |  | 1. Arterial blood gases |
|  |  | 1. Alveolar oxygen level |
|  |  | 1. **Lung clearance** |
|  | 22. Respiratory, thoracic, and mediastinal outcomes  a. Respiratory Muscle function | 1. Respiratory muscle strength |
|  |  | 1. Respiratory muscle endurance |
|  |  | 1. Maximal Expiratory Pressure (PEMax) |
|  |  | 1. Maximal Inspiratory Pressure (PIMax) |
|  | 22. Respiratory, thoracic, and mediastinal outcomes  b. Lung function | 1. Pulmonary function |
|  |  | 1. Forced expiratory volume in one second FEV1 |
|  |  | 1. Forced vital capacity (FVC) |
|  |  | 1. Maximal mid-expiratory flow (MMEF) |
|  |  | 1. Fev1 /FVC |
|  |  | 1. Forced expiratory flow between 25 and 75% of the FVC (FEF25-75%) |
|  |  | 1. Peak expiratory flow rate (PEFR) |
|  |  | 1. Inspiratory capacity (IC) |
|  |  | 1. Vital capacity (VC) |
|  |  | 1. Total lung capacity TLC |
|  |  | 1. Lung volumes |
|  |  | 1. Residual volume RV |
|  |  | 1. Lung carbon monoxide transfer factor TLCO |
|  |  | 1. Percentages of predicted spirometry values |
|  |  | 1. Ventilation inhomogeneity LCI |
|  | 22. Respiratory, thoracic, and mediastinal outcomes  c. sputum | 1. Sputum weight |
|  |  | 1. Sputum volume |
|  |  | 1. Sputum dry weight |
|  |  | 1. Change in sputum production |
|  |  | 1. In vitro mucociliary transport |
|  |  | 1. Sputum microbiology |
|  |  | 1. Secretion purulence |
|  |  | 1. Sputum Viscosity |
|  |  | 1. Sputum Elasticity |
|  |  | 1. In vitro sputum cough clearability |
|  |  | 1. The contact angle of sputum |
|  |  | 1. Secretion adhesiveness |
|  |  | 1. Sputum cell count |
|  |  | 1. In vivo mucociliary transport |
|  |  | 1. Secretion surface properties and appearance |
|  |  | 1. Sputum colour |
|  |  | 1. **Ease of sputum clearance** |
|  |  | 1. **Effect of sputum on quality of life** |
|  |  | 1. **Patient reported sputum characteristics** |
|  | 22. Respiratory, thoracic, and mediastinal outcomes d. patient reported symptoms | 1. **General patient reported symptoms** |
|  |  | 1. Breathlessness |
|  |  | 1. Number of coughs |
|  |  | 1. Cough symptoms |
|  |  | 1. Fatigue |
|  |  | 1. Change in respiratory symptoms |
|  |  | 1. Sputum symptoms |
|  |  | 1. **Perceived feeling of chest clarity** |
|  |  | 1. **Wheeze and chest rattling** |
|  |  | 1. **Pain** |
|  |  | 1. **Fever** |
|  |  | 1. **Haemoptysis** |
| Life impact | 25. Physical functioning | 1. Physical activity and fitness level/ patient reported |
|  |  | 1. Functional Exercise capacity |
|  |  | 1. Six-minute walk distance |
|  |  | 1. Maximum exercise tolerance |
|  |  | 1. Endurance walk capacity |
|  |  | 1. Maximal treadmill exercise capacity |
|  |  | 1. Energy cost in walking |
|  |  | 1. Sleep quality |
|  |  | 1. **Daily function ADL** |
|  |  | 1. **Level of disability** |
|  | **26. Social functioning** | 1. **General Social functioning** |
|  |  | 1. **Family and friends** |
|  |  | 1. **Going out** |
|  |  | 1. **Speaking** |
|  |  | 1. **Travel** |
|  | **27. Role functioning** | 1. **Work** |
|  |  | 1. **Caring for children** |
|  | 28. Emotional functioning/wellbeing | 1. **Emotional functioning and wellbeing** |
|  |  | 1. Anxiety and depression |
|  |  | 1. Confidence and self-efficacy |
|  |  | 1. **Cough stigma and social embarrassment** |
|  |  | 1. **Ability to cope** |
|  | 29. Cognitive functioning | 1. Cognitive loss |
|  | 30. Global quality of life | 1. Health-related quality of life (HRQoL) |
|  |  | 1. Cough-related quality of life |
|  |  | 1. Quality adjusted life years QALYS |
|  | 31. Perceived health status | 1. General health status |
|  | 32. Delivery of care  a. patient reported experience | 1. Patient preference |
|  |  | 1. Participant satisfaction |
|  |  | 1. Adherence to treatment |
|  |  | 1. Acceptability and Tolerance of treatment |
|  |  | 1. Comfort of technique |
|  |  | 1. Perceived benefits obtained |
|  |  | 1. General perceptions regarding interventions |
|  |  | 1. Perceived treatment effectiveness |
|  |  | 1. Illness perception |
|  |  | 1. Self-rated ability to manage bronchiectasis |
|  | 32. Delivery of care  b. intervention monitored parameters | 1. Number of sets performed during session |
|  |  | 1. Symptoms developed during intervention |
|  | **Delivery of care**  **c. Feasibility and burden of treatment** | 1. **Time burden** |
|  |  | 1. **Technique difficulty** |
|  |  | 1. **Cost of treatment** |
|  |  | 1. **Inability to perform CPT in public spaces** |
| Resource use | 35. Hospital admission | 1. Number of urgent hospital admissions |
|  |  | 1. Number of inpatient hospital days |
|  |  | 1. Number of ICU admissions |
|  |  | 1. Number of ICU days |
|  | Use of healthcare resources | 1. Number of urgent/unplanned outpatient visits |
|  |  | 1. Self-rated healthcare use |
|  | 36. Need for further intervention | 1. Need for invasive mechanical ventilation. |
|  |  | 1. Antibiotics and medication use |
| Adverse events/effects | 38. Adverse events/effects | 1. Side effects |
|  |  | 1. **Development of Urinary incontinence** |
|  |  | 1. **Development of Gastric reflux GORD** |
|  |  | 1. **Development of Postnasal drip (sinusitis)** |

**Appendix 3: Delphi study Round 1 questionnaire**

1. **Which of the following best describes you?**
2. Adult person with bronchiectasis
3. Caregiver of an adult person with bronchiectasis
4. Physiotherapist / physical therapist clinician
5. Physiotherapist / physical therapist researcher
6. Researcher/ academic and not a physiotherapist (physician or other healthcare professional)
7. Policy maker
8. Industry representative
9. Other (please specify)

**Basic information (patients)**

1. Age
2. Sex
3. Country of residence
4. Highest level of Education
   1. Primary education
   2. Secondary education
   3. University education
   4. Other
5. Years since diagnosis with bronchiectasis (insert approximate number OR I don't know)
6. Main healthcare resource of bronchiectasis care
7. Primary (GP)
8. Secondary care (hospital clinic)
9. I do not attend a healthcare professional
10. Other than medication (oral antibiotics or steroids), I have tried the following treatments, performed by myself or by a specialist (select all that applies)
11. Airway clearance breathing techniques (e.g., active cycle of breathing techniques, autogenic drainage, huffing)
12. Airway clearance devices (e.g., PEP, Flutter, Aerobika, Acapella, BubblePEP, High-Frequency Chest Wall Oscillation Vest, Airway clearance vest, Smartvest, AirPhysio, Lung flute, RC Cornet, Intrapulmonary percussive ventilation IPV))
13. Airway clearance using body positions (e.g.: postural drainage, gravity assisted drainage, ELTGOL)
14. Airway clearance using manual techniques (e.g.: manual percussion, shaking, vibration)
15. Self-management advice, and education provided by a physiotherapy specialist
16. Pulmonary rehabilitation program
17. Exercise and physical activity
18. Mucoactive drugs (e.g., normal saline, mannitol, hypertonic saline
19. Inhaled therapy (e.g.: inhaled bronchodilation, inhaled antibiotics
20. Other (please specify)
21. I received physiotherapy provided by a specialist while hospitalised (e.g., breathing techniques, positioning, manual therapy, devices, exercise)
22. Number of exacerbations (flare ups or infections) requiring change in medication during last 12 months
23. Number of antibiotic courses for bronchiectasis during last 12 months
24. Number of hospital admissions related to bronchiectasis last 12 months
25. Number of ICU admissions related to bronchiectasis last 12 months
26. Number of unplanned healthcare or doctor visits related to bronchiectasis last 12 months
27. I have /I am currently a participant in bronchiectasis treatment research

**Basic information (physiotherapists and researchers)**

1. Sex
2. Country of residence
3. Years of experience
4. Years of experience treating persons with bronchiectasis
5. Primary employment setting (select all that applies)
6. Hospital /inpatient
7. Hospital/outpatient
8. Private physiotherapy clinic
9. Community physiotherapy service
10. Research /academic institution
11. Other (specify)
12. Highest level of education/ qualification:
13. Undergraduate
14. Postgraduate (MSc or PG diploma)
15. PhD or professional doctorate degree
16. Specialised qualification in respiratory or cardiopulmonary physiotherapy (MSc, PG diploma, PhD)
17. Do you regularly see bronchiectasis patients?
18. Yes
19. No
20. On average, how many Bronchiectasis patients you see per week:
21. 1-10
22. 10-20
23. 20-30
24. 30-40
25. 40-50
26. >50
27. Research activity (select all that applies)
28. Involved in designing research
29. Involved in conducting research
30. Involved in systematic reviews
31. Involved in developing guidelines
32. Involved in clinical audit or quality control and improvement
33. Predominantly working on research
34. Not involved in research

**Instructions**

Here we are providing a list of outcomes that will have varying degrees of relevance to you depending on your experience with bronchiectasis. Please report how important the following points are to you. **Please rate each item form 1 (not important at all) to 9 (extremely important).**

Physiotherapy (also called physical therapy) may include any of the following, performed by patients at home or by a specialist in clinic:

- Airway clearance techniques (e.g., Active cycle of breathing, autogenic drainage, Huffing)
- Airway clearance devices (e.g., PEP, Flutter, Aerobika, BubblePEP, High-Frequency Chest Wall Oscillation vest, AirPhysio, Lung flute)
- Airway clearance using positioning (e.g.: postural drainage)
- Airway clearance using manual techniques (e.g.: manual percussion, shaking, vibration)
- Pulmonary rehabilitation program
- Exercise and activity
- Self-management advice and education

| **Outcome** | **How important is it to measure this outcome in research about physiotherapy for bronchiectasis?** | | | | | | | | | |
| --- | --- | --- | --- | --- | --- | --- | --- | --- | --- | --- |
| 1. **Exacerbation**   Chest infections or flare ups that require medical attention | Limited importance | | | Important but not critical | | | Critical | | | No opinion |
|  | 1 | 2 | 3 | 4 | 5 | 6 | 7 | 8 | 9 |  |
| 1. **Disease progression**   If the disease is getting worse in terms of lung damage | Limited importance | | | Important but not critical | | | Critical | | | No opinion |
|  | 1 | 2 | 3 | 4 | 5 | 6 | 7 | 8 | 9 |  |
| 1. **Clinical judgement of disease severity**   Medical judgement of how severe the condition is based on clinical assessments | Limited importance | | | Important but not critical | | | Critical | | | No opinion |
|  | 1 | 2 | 3 | 4 | 5 | 6 | 7 | 8 | 9 |  |
| 1. **Development of bacterial or viral infection**   Detection of a microorganism infection during or after physiotherapy treatment period in a research study | Limited importance | | | Important but not critical | | | Critical | | | No opinion |
|  | 1 | 2 | 3 | 4 | 5 | 6 | 7 | 8 | 9 |  |
| 1. **Development of airway inflammation**   Occurrence of immune system inflammatory response during or after physiotherapy treatment period in a research study | Limited importance | | | Important but not critical | | | Critical | | | No opinion |
|  | 1 | 2 | 3 | 4 | 5 | 6 | 7 | 8 | 9 |  |
|  | 1 | 2 | 3 | 4 | 5 | 6 | 7 | 8 | 9 |  |
| 1. **Body composition**   Body weight or mass may change due to exercise, nutrition, and inflammation | Limited importance | | | Important but not critical | | | Critical | | | No opinion |
|  | 1 | 2 | 3 | 4 | 5 | 6 | 7 | 8 | 9 |  |
| 1. **Blood Oxygen and carbon dioxide levels**   Oxygen and carbon dioxide levels in blood haemoglobin | Limited importance | | | Important but not critical | | | Critical | | | No opinion |
|  | 1 | 2 | 3 | 4 | 5 | 6 | 7 | 8 | 9 |  |
| 1. **Physical/Muscle strength**   Change in strength and endurance of skeletal muscles of arms, legs, or core | Limited importance | | | Important but not critical | | | Critical | | | No opinion |
|  | 1 | 2 | 3 | 4 | 5 | 6 | 7 | 8 | 9 |  |
| 1. **Sputum amount**   Quantity of sputum expectorated during or after physiotherapy treatment, e.g., weight or volume. | Limited importance | | | Important but not critical | | | Critical | | | No opinion |
|  | 1 | 2 | 3 | 4 | 5 | 6 | 7 | 8 | 9 |  |
| 1. **Change in sputum amount** Change from usual sputum production as reported by patient or measured by clinician as: increased, decreased, or the same | Limited importance | | | Important but not critical | | | Critical | | | No opinion |
|  | 1 | 2 | 3 | 4 | 5 | 6 | 7 | 8 | 9 |  |
| 1. **Biophysical sputum characteristics**   Laboratory testing of sputum characteristics like viscosity, adherence, elasticity etc. | Limited importance | | | Important but not critical | | | Critical | | | No opinion |
|  | 1 | 2 | 3 | 4 | 5 | 6 | 7 | 8 | 9 |  |
| 1. **Sputum purulence / colour**   Change in the colour of expectorated sputum samples from clear to darker colours | Limited importance | | | Important but not critical | | | Critical | | | No opinion |
|  | 1 | 2 | 3 | 4 | 5 | 6 | 7 | 8 | 9 |  |
| 1. **Lung function**   Reflects amount and difficulty of air inhaled and exhaled from lungs during breathing, e.g., spirometry measurements | Limited importance | | | Important but not critical | | | Critical | | | No opinion |
|  | 1 | 2 | 3 | 4 | 5 | 6 | 7 | 8 | 9 |  |
| 1. **Respiratory muscle function**   Strength and endurance of inspiration and expiration muscles | Limited importance | | | Important but not critical | | | Critical | | | No opinion |
|  | 1 | 2 | 3 | 4 | 5 | 6 | 7 | 8 | 9 |  |
| 1. **Breathlessness**   Feeling of difficulty in breathing or chest tightness | Limited importance | | | Important but not critical | | | Critical | | | No opinion |
|  | 1 | 2 | 3 | 4 | 5 | 6 | 7 | 8 | 9 |  |
| 1. **Cough**   Number of coughs or cough bouts per day, cough nature, and daytime variation | Limited importance | | | Important but not critical | | | Critical | | | No opinion |
|  | 1 | 2 | 3 | 4 | 5 | 6 | 7 | 8 | 9 |  |
| 1. **Fatigue**   Feeling tired and having no or low energy | Limited importance | | | Important but not critical | | | Critical | | | No opinion |
|  | 1 | 2 | 3 | 4 | 5 | 6 | 7 | 8 | 9 |  |
| 1. **Perceived feeling of chest congestion** Patients' feeling that chest is congested or clear from sputum | Limited importance | | | Important but not critical | | | Critical | | | No opinion |
|  | 1 | 2 | 3 | 4 | 5 | 6 | 7 | 8 | 9 |  |
| 1. **Ease of sputum clearance**   Patient-reported measure of how easy it is to remove sputum | Limited importance | | | Important but not critical | | | Critical | | | No opinion |
|  | 1 | 2 | 3 | 4 | 5 | 6 | 7 | 8 | 9 |  |
| 1. **Wheeze and chest rattling**   Rattling, crackling, or wheezing chest sounds reported by patient | Limited importance | | | Important but not critical | | | Critical | | | No opinion |
|  | 1 | 2 | 3 | 4 | 5 | 6 | 7 | 8 | 9 |  |
| 1. **Pain**   Feeling of muscle, joints, or body soreness and achiness that it hurts to move | Limited importance | | | Important but not critical | | | Critical | | | No opinion |
|  | 1 | 2 | 3 | 4 | 5 | 6 | 7 | 8 | 9 |  |
| 1. **Coughing up blood (Haemoptysis)**   Episodes of coughing up blood or blood traces in sputum | Limited importance | | | Important but not critical | | | Critical | | | No opinion |
|  | 1 | 2 | 3 | 4 | 5 | 6 | 7 | 8 | 9 |  |
| 1. **Physical functioning** General evaluation of ability to function physically, e.g., moving, doing daily activities, walking, sleeping. | Limited importance | | | Important but not critical | | | Critical | | | No opinion |
|  | 1 | 2 | 3 | 4 | 5 | 6 | 7 | 8 | 9 |  |
| 1. **Physical activity and fitness level**   Evaluation of how fit a person feels, usually by evaluating walking distance and ability to perform aerobic activities | Limited importance | | | Important but not critical | | | Critical | | | No opinion |
|  | 1 | 2 | 3 | 4 | 5 | 6 | 7 | 8 | 9 |  |
| 1. **Functional Exercise capacity/exercise tolerance**   Ability to perform a certain functional exercise (e.g., field walking distance, speed, steps, strength, balance) under controlled conditions | Limited importance | | | Important but not critical | | | Critical | | | No opinion |
|  | 1 | 2 | 3 | 4 | 5 | 6 | 7 | 8 | 9 |  |
| 1. **Vital signs variability**   Changes in heart rate, respiratory rate, and blood pressure at rest, during, and while recovering from exercise | Limited importance | | | Important but not critical | | | Critical | | | No opinion |
|  | 1 | 2 | 3 | 4 | 5 | 6 | 7 | 8 | 9 |  |
| 1. **Sleep quality**   Ability to fall asleep, stay sleeping, and having a good duration and quality of sleep. | Limited importance | | | Important but not critical | | | Critical | | | No opinion |
|  | 1 | 2 | 3 | 4 | 5 | 6 | 7 | 8 | 9 |  |
| 1. **Activities of daily living**   Ability to do daily activities like cleaning, shopping, laundry, driving, walking | Limited importance | | | Important but not critical | | | Critical | | | No opinion |
|  | 1 | 2 | 3 | 4 | 5 | 6 | 7 | 8 | 9 |  |
| 1. **Level of disability**   Ability that is significantly impaired relative to the usual standard of an individual or group, as assessed by a specialist | Limited importance | | | Important but not critical | | | Critical | | | No opinion |
|  | 1 | 2 | 3 | 4 | 5 | 6 | 7 | 8 | 9 |  |
| 1. **Frailty**   Aging-related syndrome of physical function decline, characterized by marked vulnerability to adverse health outcomes | Limited importance | | | Important but not critical | | | Critical | | | No opinion |
|  | 1 | 2 | 3 | 4 | 5 | 6 | 7 | 8 | 9 |  |
| 1. **General Social functioning**   Ability to get involved in social functioning activities, e.g., relationship with family and friends, social events, public speech, and travel | Limited importance | | | Important but not critical | | | Critical | | | No opinion |
|  | 1 | 2 | 3 | 4 | 5 | 6 | 7 | 8 | 9 |  |
| 1. **Role functioning**   Ability to perform according to role in life, e.g., being a spouse, parent, carer, professional. | Limited importance | | | Important but not critical | | | Critical | | | No opinion |
|  | 1 | 2 | 3 | 4 | 5 | 6 | 7 | 8 | 9 |  |
| 1. **Work**   Ability to work, work adjustments, and time off work | Limited importance | | | Important but not critical | | | Critical | | | No opinion |
|  | 1 | 2 | 3 | 4 | 5 | 6 | 7 | 8 | 9 |  |
| 1. **Emotional functioning and wellbeing** General evaluation of emotional and psychological wellbeing | Limited importance | | | Important but not critical | | | Critical | | | No opinion |
|  | 1 | 2 | 3 | 4 | 5 | 6 | 7 | 8 | 9 |  |
| 1. **Anxiety**   Feeling of unease, worry or fear | Limited importance | | | Important but not critical | | | Critical | | | No opinion |
|  | 1 | 2 | 3 | 4 | 5 | 6 | 7 | 8 | 9 |  |
| 1. **Depression**   Feeling "low" or feeling "blue" or having no desire for living | Limited importance | | | Important but not critical | | | Critical | | | No opinion |
|  | 1 | 2 | 3 | 4 | 5 | 6 | 7 | 8 | 9 |  |
| 1. **Confidence and self-efficacy**   Confidence of an individual to perform home treatment and self-management | Limited importance | | | Important but not critical | | | Critical | | | No opinion |
|  | 1 | 2 | 3 | 4 | 5 | 6 | 7 | 8 | 9 |  |
| 1. **Cough stigma and social embarrassment** social embarrassment and stigma, mainly related to cough in public | Limited importance | | | Important but not critical | | | Critical | | | No opinion |
|  | 1 | 2 | 3 | 4 | 5 | 6 | 7 | 8 | 9 |  |
| 1. **Ability to cope**   Ability to adjusting to or tolerating while you try to keep your positive self-image and emotional equilibrium | Limited importance | | | Important but not critical | | | Critical | | | No opinion |
|  | 1 | 2 | 3 | 4 | 5 | 6 | 7 | 8 | 9 |  |
| 1. **Cognitive loss**   Effect on mental and cognitive functioning | Limited importance | | | Important but not critical | | | Critical | | | No opinion |
|  | 1 | 2 | 3 | 4 | 5 | 6 | 7 | 8 | 9 |  |
| 1. **Health-related quality of life (HRQoL)**   An overall measure how a person's health affects their life and general wellbeing | Limited importance | | | Important but not critical | | | Critical | | | No opinion |
|  | 1 | 2 | 3 | 4 | 5 | 6 | 7 | 8 | 9 |  |
| 1. **Effect of respiratory symptoms on quality of life**   How symptoms (like cough, sputum, breathlessness, fatigue) are affecting life in general | Limited importance | | | Important but not critical | | | Critical | | | No opinion |
|  | 1 | 2 | 3 | 4 | 5 | 6 | 7 | 8 | 9 |  |
| 1. **Patient preference**   If the patient prefers a specific physiotherapy treatment | Limited importance | | | Important but not critical | | | Critical | | | No opinion |
|  | 1 | 2 | 3 | 4 | 5 | 6 | 7 | 8 | 9 |  |
| 1. **Participant satisfaction**   Level of patient's satisfaction with physiotherapy treatment | Limited importance | | | Important but not critical | | | Critical | | | No opinion |
|  | 1 | 2 | 3 | 4 | 5 | 6 | 7 | 8 | 9 |  |
| 1. **Adherence to treatment**   Measuring if the patient is applying the physiotherapy program using the correct method and required number of applications | Limited importance | | | Important but not critical | | | Critical | | | No opinion |
|  | 1 | 2 | 3 | 4 | 5 | 6 | 7 | 8 | 9 |  |
| 1. **Acceptability and Tolerance of treatment**   General perception of usefulness, ease of understanding of instructions, ease of performance, degree of tiredness, and discomfort | Limited importance | | | Important but not critical | | | Critical | | | No opinion |
|  | 1 | 2 | 3 | 4 | 5 | 6 | 7 | 8 | 9 |  |
| 1. **Perceived benefits of physiotherapy**   Benefits obtained from physiotherapy treatment according to patient | Limited importance | | | Important but not critical | | | Critical | | | No opinion |
|  | 1 | 2 | 3 | 4 | 5 | 6 | 7 | 8 | 9 |  |
| 1. **Illness perception**   Level of illness understanding including timeline, consequences, personal control, and treatment options | Limited importance | | | Important but not critical | | | Critical | | | No opinion |
|  | 1 | 2 | 3 | 4 | 5 | 6 | 7 | 8 | 9 |  |
| 1. **Self-rated ability to manage bronchiectasis**   Person's ability to manage bronchiectasis based on their own judgement | Limited importance | | | Important but not critical | | | Critical | | | No opinion |
|  | 1 | 2 | 3 | 4 | 5 | 6 | 7 | 8 | 9 |  |
| 1. **Feasibility and burden of treatment**   Evaluation of feasibility or burden of physiotherapy, based on cost, time, and difficulty aspects | Limited importance | | | Important but not critical | | | Critical | | | No opinion |
|  | 1 | 2 | 3 | 4 | 5 | 6 | 7 | 8 | 9 |  |
| 1. **Treatment Time burden**   Is the time required to apply physiotherapy per day a burden on the patient? | Limited importance | | | Important but not critical | | | Critical | | | No opinion |
|  | 1 | 2 | 3 | 4 | 5 | 6 | 7 | 8 | 9 |  |
| 1. **Technique difficulty**   How difficult is it to apply the physiotherapy technique or exercise, or use a device | Limited importance | | | Important but not critical | | | Critical | | | No opinion |
|  | 1 | 2 | 3 | 4 | 5 | 6 | 7 | 8 | 9 |  |
| 1. **Cost of treatment**   Including service costs, travel, device cost, hospital, and any other costs paid by patient, insurance, or healthcare | Limited importance | | | Important but not critical | | | Critical | | | No opinion |
|  | 1 | 2 | 3 | 4 | 5 | 6 | 7 | 8 | 9 |  |
| 1. **Use of healthcare resources**   Includes occasions patient needs hospitalisation, ICU, urgent outpatient visits to general practitioner, walk-in clinic, or emergency department | Limited importance | | | Important but not critical | | | Critical | | | No opinion |
|  | 1 | 2 | 3 | 4 | 5 | 6 | 7 | 8 | 9 |  |
| 1. **Antibiotics and medication use**   Antibiotics and other medication courses needed during or after physiotherapy treatment | Limited importance | | | Important but not critical | | | Critical | | | No opinion |
|  | 1 | 2 | 3 | 4 | 5 | 6 | 7 | 8 | 9 |  |
| 1. **Need for ICU admission or emergency invasive mechanical ventilation** | Limited importance | | | Important but not critical | | | Critical | | | No opinion |
|  | 1 | 2 | 3 | 4 | 5 | 6 | 7 | 8 | 9 |  |
| 1. **Development and/or progression of other conditions (e.g., pneumonia, gastric reflux, postnasal drip, urinary incontinence)** | Limited importance | | | Important but not critical | | | Critical | | | No opinion |
|  | 1 | 2 | 3 | 4 | 5 | 6 | 7 | 8 | 9 |  |
| 1. **Death from bronchiectasis complications**   Death from respiratory complications caused by bronchiectasis | Limited importance | | | Important but not critical | | | Critical | | | No opinion |
|  | 1 | 2 | 3 | 4 | 5 | 6 | 7 | 8 | 9 |  |
| 1. **Death from any cause**   Death from any cause during or after study completion | Limited importance | | | Important but not critical | | | Critical | | | No opinion |
|  | 1 | 2 | 3 | 4 | 5 | 6 | 7 | 8 | 9 |  |

1. Are there any other outcomes that you think are important to measure and report in trials? If yes, please write them below:
2. We would like to contact you by email to provide you with responses to this round and to forward you the round 2 questionnaire. We would appreciate it if you provided your email below:

**END OF QUESTIONNAIRE**

**Thank you very much for your participation.** You will receive round 2 questionnaire on the email you provided.

**Appendix 4: Screenshot of one item in R2 Delphi survey**
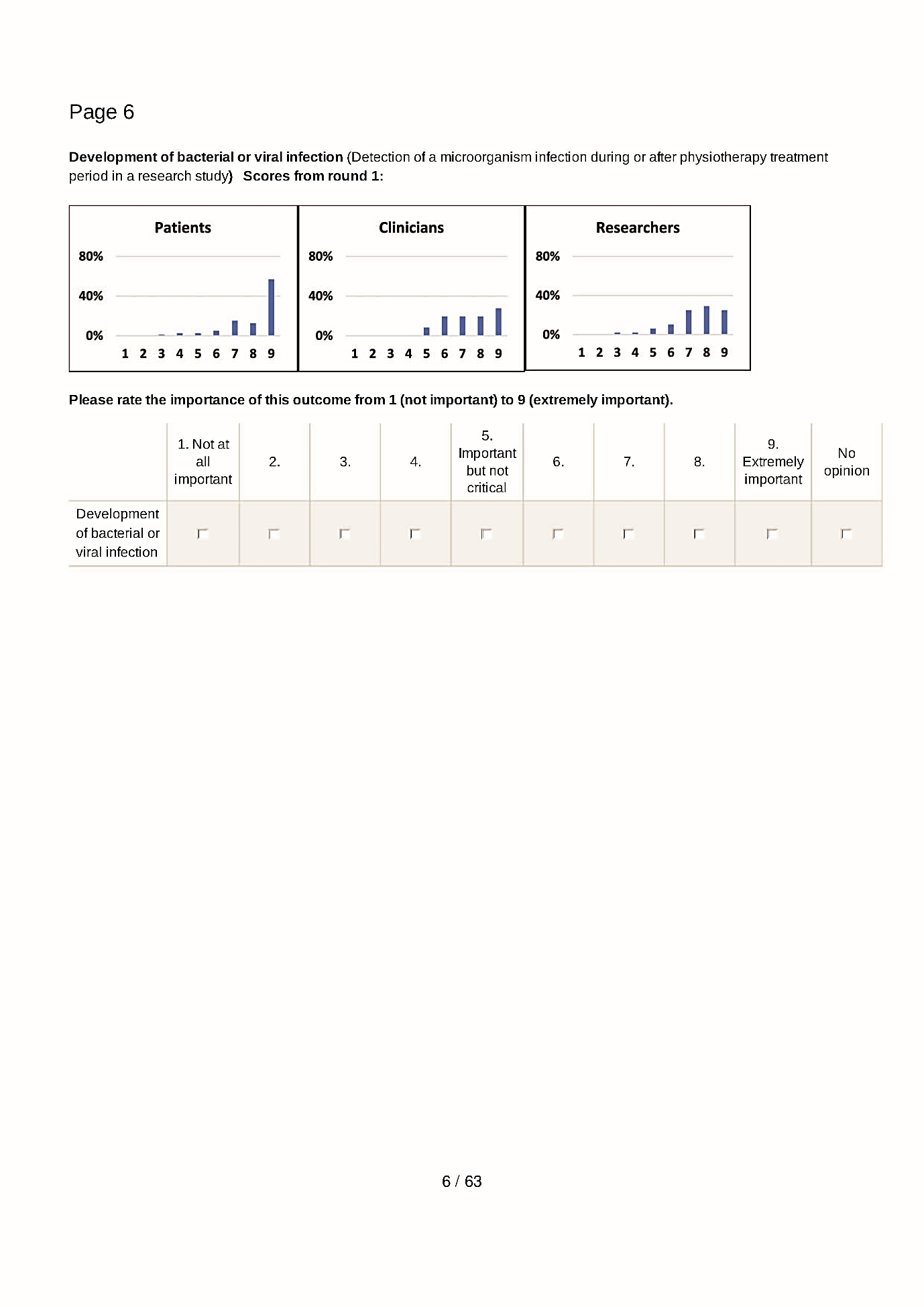


**Appendix 5: Details of the Delphi study results. The percentage of participants that scored an outcome as critical (7-9)**

| **Voting results (% extremely important 7-9)** | **Round one** | | | **Round Two** | | | **Decision** |
| --- | --- | --- | --- | --- | --- | --- | --- |
| **Outcomes** | **P** | **PT** | **R** | **P** | **PT** | **R** |  |
| 1. Exacerbation | 91% | 94% | 94% | 94% | 100% | 93% | IN |
| 1. Disease progression | 91% | 86% | 80% | 96% | 100% | 90% | IN |
| 1. Clinical judgement of disease severity | 86% | 78% | 61% | 90% | 81% | 67% | No Consensus |
| 1. Development of bacterial or viral infection | 84% | 67% | 80% | 87% | 76% | 83% | IN |
| 1. Development of airway inflammation | 78% | 69% | 73% | 91% | 71% | 63% | No Consensus |
| 1. Body composition | 54% | 44% | 49% | 47% | 33% | 30% | OUT |
| 1. Blood Oxygen and carbon dioxide levels | 73% | 50% | 37% | 85% | 38% | 30% | No Consensus |
| 1. Physical/Muscle strength | 60% | 75% | 84% | 84% | 86% | 80% | IN |
| 1. Sputum amount | 73% | 83% | 86% | 91% | 90% | 97% | IN |
| 1. Change in sputum amount | 79% | 94% | 88% | 88% | 95% | 93% | IN |
| 1. Biophysical sputum characteristics | 68% | 67% | 57% | 88% | 67% | 60% | No Consensus |
| 1. Sputum purulence / colour | 76% | 81% | 61% | 87% | 76% | 83% | IN |
| 1. Lung function | 86% | 81% | 61% | 93% | 71% | 67% | No Consensus |
| 1. Respiratory muscle function | 78% | 75% | 67% | 90% | 67% | 63% | No Consensus |
| 1. Breathlessness | 91% | 78% | 86% | 97% | 100% | 93% | IN |
| 1. Cough | 71% | 75% | 84% | 88% | 100% | 87% | IN |
| 1. Fatigue | 79% | 83% | 82% | 88% | 95% | 90% | IN |
| 1. Perceived feeling that chest is congested or clear | 80% | 69% | 78% | 88% | 90% | 67% | No Consensus |
| 1. Ease of sputum clearance | 86% | 83% | 90% | 97% | 100% | 90% | IN |
| 1. Wheeze and chest rattling | 78% | 67% | 63% | 85% | 76% | 63% | No Consensus |
| 1. Pain | 78% | 56% | 55% | 85% | 38% | 40% | No Consensus |
| 1. Coughing up blood (Haemoptysis) | 89% | 81% | 73% | 91% | 86% | 80% | IN |
| 1. Physical functioning | 83% | 83% | 78% | 96% | 100% | 97% | IN |
| 1. Physical activity and fitness level | 71% | 86% | 80% | 93% | 100% | 97% | IN |
| 1. Functional Exercise capacity/exercise tolerance | 69% | 81% | 84% | 91% | 100% | 97% | IN |
| 1. Vital signs variability | 74% | 61% | 61% | 85% | 67% | 50% | No Consensus |
| 1. Sleep quality | 71% | 58% | 63% | 87% | 71% | 57% | No Consensus |
| 1. Activities of daily living | 79% | 69% | 76% | 93% | 100% | 83% | IN |
| 1. Level of disability | 74% | 69% | 73% | 82% | 76% | 67% | No Consensus |
| 1. Frailty | 74% | 58% | 69% | 84% | 76% | 57% | No Consensus |
| 1. General Social functioning | 66% | 69% | 67% | 81% | 90% | 67% | No Consensus |
| 1. Role functioning | 66% | 64% | 71% | 84% | 81% | 60% | No Consensus |
| 1. Work | 68% | 61% | 73% | 85% | 81% | 57% | No Consensus |
| 1. Emotional, psychological functioning and wellbeing | 79% | 75% | 82% | 94% | 90% | 77% | IN |
| 1. Anxiety | 71% | 64% | 78% | 84% | 90% | 77% | IN |
| 1. Depression | 68% | 67% | 78% | 87% | 86% | 67% | No Consensus |
| 1. Confidence and self-efficacy | 76% | 72% | 82% | 93% | 95% | 80% | IN |
| 1. Cough stigma and social embarrassment | 64% | 67% | 73% | 75% | 86% | 77% | IN |
| 1. Ability to cope | 75% | 67% | 67% | 90% | 86% | 73% | IN |
| 1. Cognitive loss | 71% | 50% | 65% | 82% | 67% | 47% | No Consensus |
| 1. Health-related quality of life | 84% | 86% | 94% | 96% | 100% | 97% | IN |
| 1. Symptom-related quality of life | 89% | 92% | 94% | 99% | 95% | 97% | IN |
| 1. Patient preference | 63% | 64% | 76% | 90% | 90% | 87% | IN |
| 1. Participant satisfaction with physiotherapy | 69% | 89% | 90% | 90% | 100% | 87% | IN |
| 1. Adherence to treatment | 81% | 100% | 90% | 94% | 100% | 97% | IN |
| 1. Acceptability and Tolerance of physiotherapy | 84% | 94% | 90% | 93% | 100% | 93% | IN |
| 1. Perceived benefits of physiotherapy | 84% | 92% | 86% | 91% | 100% | 87% | IN |
| 1. Illness perception | 86% | 72% | 73% | 96% | 90% | 60% | No Consensus |
| 1. Self-rated ability to manage bronchiectasis | 83% | 86% | 80% | 97% | 100% | 83% | IN |
| 1. Feasibility and burden of physiotherapy | 75% | 83% | 78% | 88% | 95% | 87% | IN |
| 1. Treatment Time burden | 74% | 83% | 78% | 90% | 90% | 83% | IN |
| 1. Technique difficulty | 76% | 72% | 78% | 90% | 95% | 80% | IN |
| 1. Cost of treatment | 73% | 64% | 69% | 76% | 90% | 80% | IN |
| 1. Use of healthcare resources | 80% | 72% | 80% | 91% | 90% | 83% | IN |
| 1. Need for Antibiotics and medication | 88% | 81% | 73% | 90% | 90% | 80% | IN |
| 1. Need for ICU or emergency invasive mechanical ventilation | 75% | 75% | 71% | 88% | 86% | 63% | No Consensus |
| 1. Development and/or progression of other conditions | 76% | 61% | 65% | 90% | 71% | 63% | No Consensus |
| 1. Death from bronchiectasis complications | 86% | 86% | 76% | 96% | 86% | 90% | IN |
| 1. Death from any cause | 65% | 50% | 51% | 72% | 48% | 57% | No Consensus |
| 1. General health status |  |  |  | 82% | 81% | 63% | No Consensus |
| 1. Sexual function |  |  |  | 38% | 52% | 43% | OUT |
| P=Patients, PT=Physiotherapists, R=Researchers | | | | | | | |

**Appendix 6: Means distributions of participants who completed R1 only versus both rounds**

| Group | Completed R1 Mean (SD) | Completed both rounds Mean (SD) |
| --- | --- | --- |
| All | 7.74 (0.93) | 7.65 (0.97) |
| Patients | 7.48 (1.22) | 7.85 (0.95) |
| Clinicians | 7.26 (0.75) | 7.52 (1.01) |
| Researchers | 7.69 (0.63) | 7.30 (0.87) |

**Appendix 7: Results of the consensus meeting voting.**

| **Outcome** | **Professionals** | **Patients** | **ALL** | **Decision** |
| --- | --- | --- | --- | --- |
| 1. Exacerbations | 63% | 71% | 67% | OUT |
| 1. Disease progression | 38% | 83% | 57% | OUT |
| 1. Development of bacterial or viral infection | 0% | 40% | 15% | OUT |
| 1. Death from bronchiectasis complications | 0% | 14% | 7% | OUT |
| 1. Breathlessness | 50% | 71% | 60% | OUT |
| 1. Cough | 75% | 57% | 67% | OUT |
| 1. Fatigue | 71% | 75% | 71% | IN |
| 1. Ease of sputum clearance | 50% | 86% | 67% | OUT |
| 1. Coughing up blood (Haemoptysis) | 13% | 38% | 27% | OUT |
| 1. Health-related quality of life | 88% | 100% | 93% | IN |
| 1. Respiratory symptoms | 88% | 71% | 80% | IN |
| 1. Physical functioning | 88% | 100% | 93% | IN |
| 1. Activities of daily living | 88% | 86% | 87% | IN |
| 1. Emotional, psychological functioning and wellbeing | 100% | 100% | 100% | IN |
| 1. Anxiety | 38% | 38% | 36% | OUT |
| 1. Confidence and self-efficacy | 50% | 86% | 67% | OUT |
| 1. Cough stigma and social embarrassment | 13% | 29% | 20% | OUT |
| 1. Ability to cope | 0% | 86% | 43% | OUT |
| 1. Patient preference | 50% | 43% | 47% | OUT |
| 1. Participant satisfaction with physiotherapy | 13% | 29% | 20% | OUT |
| 1. Adherence to treatment | 63% | 100% | 80% | IN |
| 1. Acceptability and Tolerance of treatment | 63% | 71% | 67% | OUT |
| 1. Perceived benefits of physiotherapy | 38% | 80% | 54% | OUT |
| 1. Self-rated ability to manage bronchiectasis | 25% | 83% | 50% | OUT |
| 1. Feasibility and burden of physiotherapy | 50% | 86% | 67% | OUT |
| 1. Treatment Time burden | 57% | 43% | 50% | OUT |
| 1. Technique difficulty | 0% | 43% | 21% | OUT |
| 1. Cost of treatment | 0% | 14% | 7% | OUT |
| 1. Use of healthcare resources | 29% | 29% | 29% | OUT |
| 1. Antibiotics and medication use | 0% | 57% | 27% | OUT |
| 1. Sputum amount | 0% | 50% | 21% | OUT |
| 1. Change in sputum amount | 44% | 100% | 64% | OUT |
| 1. Sputum purulence / colour | 38% | 83% | 57% | OUT |
| 1. Physical/Muscle strength | 63% | 67% | 64% | OUT |
| 1. Physical activity and fitness level | 25% | 67% | 43% | OUT |
| 1. Functional Exercise capacity/exercise tolerance | 86% | 100% | 92% | IN |

**Appendix 8: Consensus meeting feedback results (n=12)**

| Question | Strongly agree or agree | Neither agree nor disagree | Disagree or strongly disagree |
| --- | --- | --- | --- |
| The information that the organisers provided me with in advance of the meeting was helpful. | 12 (100%) | 0 | 0 |
| I was satisfied with the process used to agree the core outcomes set on the meeting day. | 9 (75%) | 3 (75%) | 0 |
| I was satisfied with the way the meeting was facilitated. | 11 (92%) | 0 | 1 (8%) |
| I felt able to contribute to the meeting. | 11 (92%) | 1 (8%) | 0 |
| I felt comfortable in communicating my views. | 12 (100%) | 0 | 0 |
| The meeting produced a fair result. | 8 (67%) | 4 (33%) | 0 |
